# Nitrogen Deposition in the Greater Tehran Metropolitan Area

**DOI:** 10.1100/tsw.2001.389

**Published:** 2001-12-08

**Authors:** Ali Salahi, Shirin Geranfar, Soudabeh A.A. Korori

**Affiliations:** Research Institute of Forests and Rangelands, Alborz Research Center, Karaj, Iran

**Keywords:** nitrate ion (NO_3_), photochemical reaction, ammonia (NH_3_), wet deposition, acidity (pH), suspended particulated matter (SPM)

## Abstract

An investigation of air pollution in the Tehran metropolitan area between 1992–2000 indicated that there are significant amounts of nitrate ion (NO_3_), over 30 kg/ha/year, deposited as wet deposition, compared to 13 kg/ha/year in the Chitgar Parkland near the Tehran metropolitan area. The amount of NO_3_ in warm seasons is twofold that of cold seasons (see Fig. 1), and there was a significant difference between cold and warm seasons (Table 1). Annual wet deposition of ammonia (NH_3_) was 10 kg/ha/year in the Chitgar Parkland[1].

## INTRODUCTION

Human activities have recently caused the fall of acid rain and polluted precipitation, containing nitrogen oxdies (NOx) and sulfur dioxide ($SO_2$) which result from the consumption of fossil fuels, condensation of mineral materials, and oil refineries, combined with the atmospheric acids. Acid rain has destructive effects on forests, causing a decrease in organic matters of soils and increasing soil erosion as well as loss of underground water reservoirs. Acid deposition on vegetation causes their higher susceptibility to pests and pathogen factors[2].

Investigation of acid rain in the area south of Tehran indicates that annual deposition of nitrate ion ($NO_3^-$) in some southern areas of Tehran is more than 30 kg/ha/year[3], and sulfate ion ($SO_4^{2-}$) desposition in some southern areas of Tehran is approximately 300 kg/ha/year[4], compared to 80 kg/ha/year in some sampling stations of Chitgar Parkland (Fig. 2). Such huge amounts of air pollutants come down with snow and rain[5].

## EXPERIMENTAL METHOD/PROCEDURES

This experiment and study used a method of wet deposition by precipitation. For measurement and sampling, specific instruments were designed and established in different stations south of Tehran, and also on the east and west sides of Tehran. Samples were analyzed in the Analytical Chemistry Laboratory using a flame photometer for potassium and sodium, a spectrophotometer for $SO_4^{2-}$, and a titration for $NO_3^-$.

## RESULTS AND DISCUSSION

Monthly rainfall indicates that, in spite of the production of NOx in the cooling season, total nitrate is decreased over winter (Fig. 1). The reason is that production of $NO_3^-$ originates from photochemical processes, and the conditions for photochemical

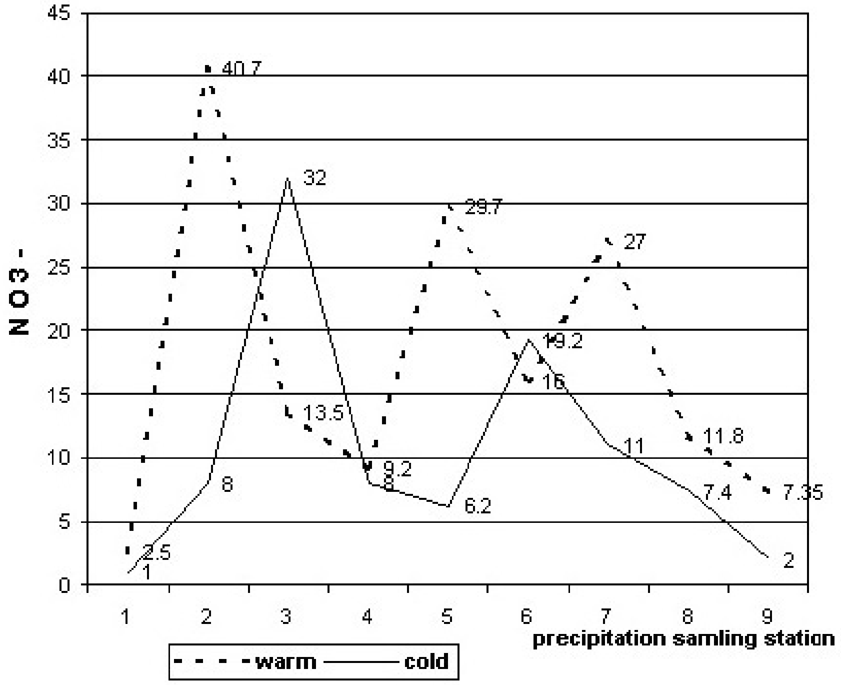

FIGURE 1. Annual wet deposition of $NO_3^-$ in kg/ha/year inside Chitgar Parkland in west greater Tehran metropolitan area during warm and cold seasons.

**TABLE 1**
**Dependent Variable: pH; Independent Variable : $NO_3^-$**

**Regression Analysis**

| Parameter | Estimate | St. Error | T-Value | Prob. |
|---|---|---|---|---|
| Intercept | 6.048 | 0.199 | 30.457 | 0.0000 |
| Slope | 0.071 | 0.035 | 2.017 | 0.0522 |

**Analysis of Variance**

| Source | S.S. | D.F. | M.F. | F | Prob. |
|---|---|---|---|---|---|
| Model | 2.240 | 1 | 2.240 | 4.07 | 0.0966 |
| Residual | 17.620 | 32 | 0.551 | | |

*Note:* R = 0.34*

reactions are not provided in winter. Therefore, production of $NO_3^-$ is decreased (Tables 2 and 3). Amounts of $NO_3^-$ as wet deposition in a number of sampling stations in Tehran was over 30 kg/ha/year, and 13 kg/ha/year for the Chitgar Parkland, 15 km west of the Tehran metropolitan area. The amount of $NO_3^-$ in warm seasons was more than twofold that of cold seasons (Fig. 1). Seasonal sampling of $NO_3^-$ showed that there was significant difference ($p = 0.01$) between cold and warm seasons (Table 3)[1]. In the cold period, increased production of $NO_3^-$ does not parallel the increased consumption of fossil fuels. This means that conditions for photochemical reactions do not occur during winter and cold seasons. Ammonia ($NH_3$) is formed as a result of the decomposition of most nitrogenous organic materials used as fertilizer and as a chemical intermediate. Evaporation from animal manure accounts for most of the emissions of $NH_3$ to the atmosphere. It is, however, the surplus of N in the farming cycle that lies at the bottom of the problem, produced as a result of industrialized farming methods based on the use of artificial fertilizer[6].

Annual wet deposition of $NH_3$ was 9 kg/ha/year (Fig. 3). Acidity (pH) of precipitation is neutralized by suspended particulate matter (SPM). However, many samples of precipitation showed acid rain (pH = 4) in Tehran. This minimum measured acidity in Tehran is 50 times as acidic as atmospheric background

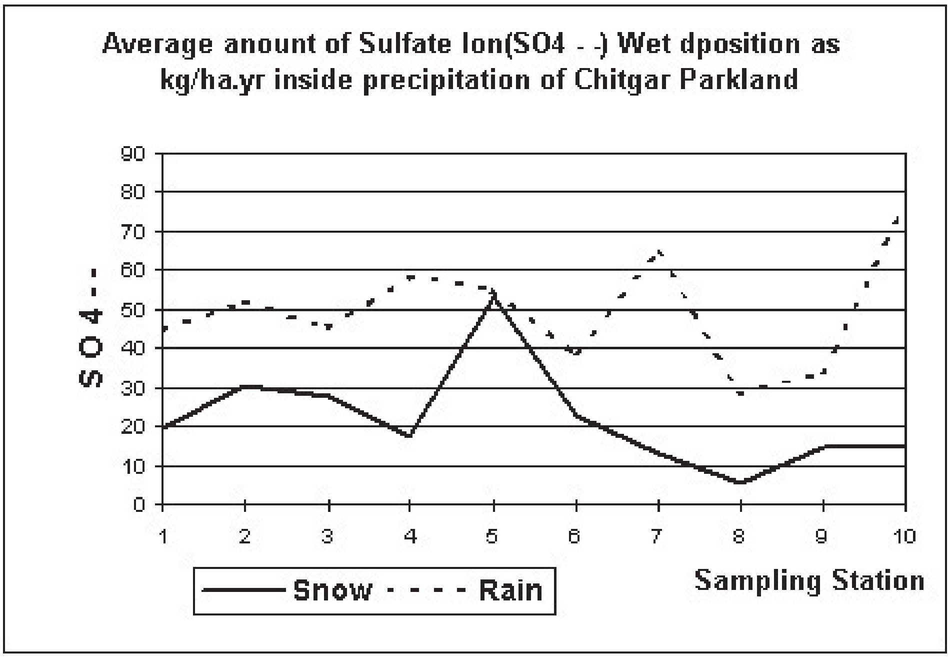

**FIGURE 2.** Average amount of $SO_4^{2-}$ wet deposition as kg/ha/year inside precipitation of Chitgar Parkland.

**TABLE 2**
**Analysis of Variance for $NO_3^-$ Related to Precipitation in Chitgar Parkland**

| K Value | Source | Degrees of Freedom | Sum of Squares | Mean Square | F Value | Prob. |
|---|---|---|---|---|---|---|
| 1 | Replication | 5 | 213.816 | 42.763 | 1.6973 | 0.1721 |
| 2 | Factor A | 2 | 50.378 | 25.189 | 0.9998 | |
| 4 | Factor B | 1 | 130.721 | 130.721 | 5.1884 | 0.0316 |
| 6 | AB | 2 | 99.369 | 49.684 | 1.9720 | 0.1602 |
| –7 | Error | 25 | 629.868 | 25.195 | | |
| | Total | 35 | 1124.152 | | | |

**TABLE 3**
**Duncan's Multiple Range Test Related to $NO_3^-$ for Different Seasons (time) of Precipitation Sampling**

| | | |
|---|---|---|
| Warm seasons | 7.551 | a |
| Cold seasons | 3.789 | b |

acidity. Analysis of variance for acidity (pH) related to precipitation in Chitgar Parkland showed that there was significant difference ($p$ = 0.01) for different blocks of precipitation sampling (Tables 4 and 5). The regression equation for the independent variable of $NO_3^-$ and dependent variable of acidity (pH) was computed as Y = 6.048 + 0.071 X (Fig. 4). The coefficient of determination was 0.34 (r = 0.34). The coefficient of determination for correlation between $NO_3^-$ and $SO_4^{2-}$ was 0.64, between $NO_3^-$ and electro-conductivity (EC) was 0.74, and between $NO_3^-$ and Na+ was 0.57[7].

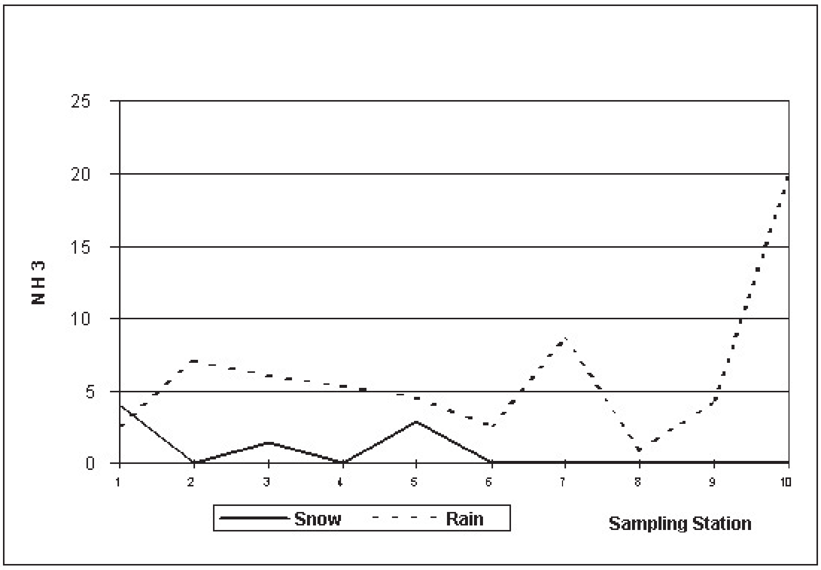

**FIGURE 3**. Average amount of $NH_3$ inside precipitation of Chitgar Parkland (kg/ha/year).

**TABLE 4**
**Analysis of Variance for Acidity (pH) Related to Precipitation in Chitgar Parkland**

| K Value | Source | Degrees of Freedom | Sum of Squares | Mean Square | F Value | Prob. |
|---|---|---|---|---|---|---|
| **1** | Replication | 5 | 4.836 | 0.967 | 2.0430 | 0.1070 |
| **2** | Factor A | 2 | 6.286 | 3.143 | 6.6390 | 0.0049 |
| **4** | Factor B | 1 | 0.912 | 0.912 | 1.9265 | 0.1774 |
| **6** | AB | 2 | 5.097 | 2.548 | 5.3830 | 0.0114 |
| **–7** | Error | 25 | 11.835 | 0.473 | | |
| | Total | 35 | 28.966 | | | |

**TABLE 5**
**Duncan's Multiple Range Test Related to pH for Different Treatment of Precipitation Sampling**

| | | |
|---|---|---|
| Near freeway | 6.788 | **a** |
| Center of Parkland | 6.488 | **ab** |
| Iner part of Parkland | 5.791 | **b** |

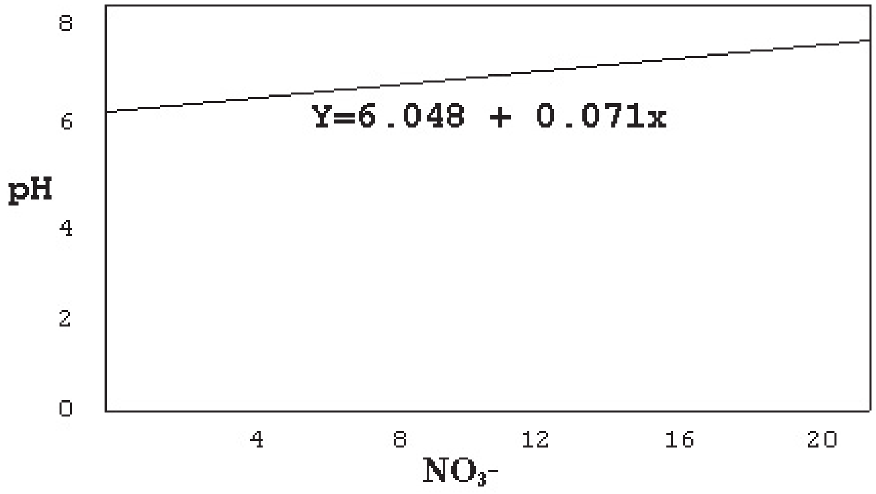

**FIGURE 4**. Regression line for pH (dependent variable) and $NO_3^-$ (independent variable) studied south of Tehran.

## CONCLUSION

The main contributor to the concentration of NOx in urban surroundings is usually road traffic, although in some cities combustion plants make a significant contribution. Animal manure consequently becomes a waste product on farms with intensive stock raising, while those concentrating on grain growing have to make up for a shortage of nutrients by using artificial fertilizer. Since so much of the feed stuff production is now dependent on the use of artificial fertilizer, it can be said that the $NH_3$ that emanates from animal manure has its origin in the artificial additives. Where farming operations are thus unbalanced, N is continually being added in the form of artificial fertilizer, and leaks out into air and water in great quantities. Hence, the joint strategy puts more emphasis on cutting down NOx emissions, and more research on health effects is needed.

## ACKNOWLEDGEMENTS

The author would like to thank Mrs. Shirin Geranfer for close cooperation, Mr. Hamid Yarmand, former head of the Research Division of Forests and Rangelands Health and Protection, and Professor Mahmoud Shariat, Head of the Environmental Health Group, Tehran University. The author also wishes to thank Mr. Vahid Moniri, Mr. Rasoul Omid, and Mr.Ebrahim Aziz–Khani, colleagues who encouraged me and helped me during research works.

| | | |
|---|---|---|
| Received: | July | 28, 2001 |
| Revised: | November | 11, 2001 |
| Accepted: | November | 12, 2001 |
| Published: | December | 8, 2001 |
